# Single detection of human bocavirus 1 with a high viral load in severe respiratory tract infections in previously healthy children

**DOI:** 10.1186/1471-2334-14-424

**Published:** 2014-07-30

**Authors:** Lili Zhou, Shouyan Zheng, Qiuyan Xiao, Luo Ren, Xiaohong Xie, Jian Luo, Lijia Wang, Ailong Huang, Wei Liu, Enmei Liu

**Affiliations:** Ministry of Education Key Laboratory of Child Development and Disorders; Key Laboratory of Pediatrics in Chongqing, CSTC2009CA5002, Chongqing International Science and Technology Cooperation Center for Child Development and Disorders, Chongqing, 400014 P.R. China; Department of Respiratory Medicine, Children's Hospital of Chongqing Medical University, Chongqing, 400014 P.R. China; Key Laboratory of Molecular Biology of Infectious Diseases, Ministry of Education, Chongqing Medical University, Chongqing, P.R. China; State Key Laboratory of Pathogens and Biosecurity, Beijing Institute of Microbiology and Epidemiology, Beijing, P.R. China

**Keywords:** HBoV1, Viral load, Single detection, Severe respiratory tract infection, Children

## Abstract

**Background:**

Human bocavirus is a newly discovered parvovirus. Multiple studies have confirmed the presence of human bocavirus1 (HBoV1) in respiratory tract samples of children. The viral load, presentation of single detection and its role as a causative agent of severe respiratory tract infections have not been thoroughly elucidated.

**Methods:**

We investigated the presence of HBoV1 by quantitative polymerase chain reaction (PCR) of nasopharyngeal aspirate specimens from 1229 children hospitalized for respiratory tract infections. The samples were analyzed for 15 respiratory viruses by PCR and 7 respiratory viruses by viral culture.

**Results:**

At least one virus was detected in 652 (53.1%) of 1229 children, and two or more viruses were detected in 266 (21.6%) children. HBoV1 was detected in 127 children (10.3%), in which 66/127 (52%) of the cases were the only HBoV1 virus detected. Seasonal variation was observed with a high HBoV1 infection rate in summer. A cutoff value of 10^7^ copies/mL was used to distinguish high and low HBoV1 viral loads in the nasopharyngeal aspirates. High viral loads of HBoV1 were noted predominantly in the absence of other viral agents (28/39, 71.8%) whereas there was primarily co-detection in cases of low HBoV1 viral loads (50/88, 56.8%). There were no differences in the clinical symptoms and severity between HBoV1 single detection and co-detection. In cases of HBoV1 single detection, the high viral load group was more prevalent among children with dyspnea and wheezing than was the low viral load group (42.9% vs. 23.7%, P = 0.036; 60.7% vs. 31.6%, P = 0.018). In clinical severity, a significant difference was recorded (25.0% vs. 5.3%, P = 0.003) between high viral load and low viral load groups. Of the HBoV1 positive patients associated with severe respiratory tract infections, 10/18 (55.6%) patients belonged to the HBoV1 high viral load group, and 7/10 (70%) patients had cases of HBoV1 single detection.

**Conclusions:**

HBoV1 at a high viral load is not frequently found in co-detection with other respiratory viruses, and a single detection with a high viral load could be an etiological agent of severe respiratory tract infections.

**Electronic supplementary material:**

The online version of this article (doi:10.1186/1471-2334-14-424) contains supplementary material, which is available to authorized users.

## Background

Human bocavirus (HBoV) was first described in 2005 in nasopharyngeal aspirates (NPAs) of children with respiratory tract infections (RTI). Although the prevalence of HBoV in humans has been studied in some regions, it has not been well addressed globally [1]. Nearly all prevalence studies have been done on respiratory tract secretions from patients (predominantly children) with RTI, and prevalence rates of between 1.5% and 19% have been observed [2].

Our current knowledge of HBoV1 infection suggests that the virus is sometimes a passenger and sometimes a pathogen in acute respiratory tract disease. Allander suggested a model for HBoV infection in which high viral loads are potentially associated with respiratory symptoms, and low viral loads indicate asymptomatic shedding [3]. Jacques reported that HBoV at a high viral load could be an etiological agent of respiratory tract disease [4]. The patients positive only for HBoV predominantly constituted the high viral load group, whereas most of the HBoV-positive patients with infection caused by other respiratory viruses belonged to the low viral load group [4–6]. Martin reported that HBoV positivity did not consistently correspond with the onset of respiratory illness, and its load did not correlate with the severity of illness [7]. Recent results obtained by quantitative real-time PCR suggest that high HBoV viral loads (defined as > 10^6^copies/mL) are frequently present as the sole viral finding for children admitted for RTI [4, 5, 8]. However, the role of HBoV as a causative agent in severe respiratory tract infections (SRTI) is unclear.

HBoV infection has recently attracted increasing attention all over the world. The incidence and clinical presentation of this infection varies widely, and often involves co-infection with other potential pathogens [9]. Respiratory syncytial virus (RSV) was the most prevalent pathogen associated with HBoV in all the studies [2, 10–13]. Such characteristics have led to debate over the role of HBoV as a true pathogen. Therefore, additional evidence and studies are needed throughout the world to gain a better understanding of this virus. We investigated whether HBoV1 at a high viral load increases the severity of RTI and whether co-detection with HBoV1 and another respiratory virus or viruses increases the severity of concurrent viral detection. We study the prevalence of HBoV1 and the genome of the HBoV1 load in respiratory tract specimens from children hospitalized for RTI to investigate the association between HBoV1 detection and SRTI. This article might increase our understanding of the role of HBoV1 in SRTI.

## Methods

### Study subjects and sample collection

Between December 2009 and August 2013, we recruited 1229 children with RTI (the clinical systems included cough, expectoration, tachypnea, and wheezing) from the Department of Respiratory Medicine at the Children’s Hospital of Chongqing Medical University in China. NPAs were collected when the patients were admitted to our department. The specimens were kept at 4°C for a maximum of 4 h and stored at -80°C until further processing. This study was authorized by the Ethics Committee of the Children’s Hospital of Chongqing Medical University. The guardians of the patients signed informed consent forms for participation in this study and for the publication of individual clinical details.

### Diagnosis of SRTI

SRTI was assessed according to respiratory failure confirmed by an abnormal blood gas analysis result (based on the potential of hydrogen, partial pressure CO2, partial pressure O2, an oxygen saturation level of approximately 90% or less and the need for oxygen therapy) or by being a patient in intensive care unit (ICU) for mechanical ventilation treatment [14].

### Virus isolation

Viral culture was performed for 7 respiratory viruses for all samples: adenovirus (ADV), influenza virus A and B (IVA and IVB), parainfluenza virus types 1–3 (PIV1–3) and RSV [15]. Human epidermoid carcinoma Hep-2 cells were maintained in Dulbecco Modified Eagle’s Medium (DMEM) supplemented with 10% fetal bovine serum (FBS), 50 U/mL penicillin and 50-g/mL streptomycin (from Invitrogen, Carlsbad, CA). The cells were seeded and cultured to 70 to 80% confluence, and the clinical specimens were inoculated in the Hep-2 cells and cultured for 2 h. Next, the inoculum was aspirated and the Hep-2 cells were washed twice with phosphate buffered saline (PBS) and re-fed with fresh DMEM supplemented with 2% FBS. The cells were scraped when the cytopathic effect (CPE) involved at least 75% of the Hep-2 cell monolayer, after which the virus-cell suspensions were collected, aliquoted and stored at -80°C. If the cells showed no CPE after being cultured for 7 days, they were scraped, and the cell suspension was collected for a second inoculation. For CPE positive samples, virus nucleic acids were extracted from the virus-cell suspensions and nested PCR was used to confer what virus(es) were there (the methods were mentioned as follows).

### Means of diagnosis for 15 respiratory viruses

The viral DNA and RNA were extracted from 200 μL aliquots of the NPA samples using the QIAampMinElute Virus Spin kit (Qiagen, Hilden, Germany). The RNA was applied as the template for cDNA synthesis using the SuperScript III First-Strand Synthesis System (Invitrogen, California, USA). The DNA and RNA extractions and cDNA products were used for subsequent testing of 15 respiratory viruses. All the samples were analyzed using a commercial detection kit (TaKaRa Biotechonology, Dalian, China and Applied Biosystems, California, USA), according to the manufacturer’s instructions. The 15 respiratory viruses detected were as follows [3, 16–18]: RSV subtypes A and B (RSVA, RSVB); IVA, IVB, influenza virus C (IVC); coronaviruses (CoV-229E, CoV-OC43), metapneumovirus (MPV), PIV1-4, rhinovirus (RV), HBoV1 and ADV. Real-time PCR was used to detect RV and HBoV1. Nested PCR assays were used to detect RSVA, RSVB, IVA, IVB, IVC, CoV-229E, CoV-OC43, MPV, PIV 1–4 and ADV.

### Real-time fluorescence quantitative PCR for HBoV1

Amplification of HBoV1 DNA by real-time quantity PCR was performed with the NS1 primers and probe [19, 20]. The plasmid amplified target fragment was cloned into the pMD19-T vector (TaKaRa Biotechonology, Dalian, China) and verified by sequencing. Plasmid DNA concentrations were detected with an ND-1000 spectrophotometer (Nanodrop). Real-time fluorescence quantitative PCR was carried out in a total reaction volume of 20 μL consisting of 10 μL of TaqMan Universal Master Mix (Applied Biosystems, California, USA), 0.6 μL (0.6 mM) of each primer, 0.6 μL (0.3 mM) of the probe, 2 μL of template and 6.2 μL of double-distilled water. The real-time PCR thermal cycling reaction and quantitative measurement were performed in a StepOne Real-Time PCR instrument (Applied Biosystems, California, USA) using the following conditions: one cycle at 50°C for 2 min, one cycle at 95°C for 10 min, 40 cycles at 95°C for 15 s, and one cycle at 60°C for 1 min. Each run included plasmid and negative controls. Standard precautions were taken throughout the PCR process to avoid cross-contamination. Negative controls and serial dilutions of the positive controls were included in every PCR assay.

#### Statistical analyses

The statistical analyses were carried out using the SPSS 17.0 software package. The categorical variables were compared using the Chi-square test, and the continuous variables were compared using Student’s *t*-test or the nonparametric Mann–Whitney *U*-test. P-values <0.05 were considered to be significant.

## Results

### Demographic and viral findings in children with RTI

During the successive 3.5-year period, 1229 patients with RTI were included in this study. The median age of the children was 8 months, varying from 1 month to 203 months, of which 63.1% of the patients were under 12 months (41.9% patients ≤6 months), 15.6% of the patients were between 1 and 2 years, 15.2% of the patients were between 2 and 5 years, whereas 6.0% of the patients were older than 5 years of age. Among the patients, there were 834 boys and 395 girls, and the sex ratio was 2.1:1. A potential viral pathogen was identified in 652 (53.1%) children (Table 1). For 1229 patients, PCR screening was performed for all 15 viruses and viral culture was performed for 7 viruses. The PCR results were positive for ≥1 virus in 652 (53.1%) children. The viral culture results were positive for ≥1 virus in 288 (23.4%) children. Notably, 266 (21.6%) of the patients had positive test results for ≥ 2 viruses.

**Table 1:** **Viral etiology of respiratory infection in 1229 children with respiratory tract infections**

Viruses	No. (%) of children infected with virus	No. (%) of children infected with virus as sole agent
Rhinovirus	318 (25.9)	105 (8.5)
Respiratory syncytial virus A and B	197 (16.0)	112 (9.1)
Human bocavirus 1	127 (10.3)	66 (5.4)
Parainfluenza virus types 1–4	44 (3.6)	12 (1.0)
Adenovirus	88 (7.2)	47 (3.8)
Influenza A, B, and C viruses	34 (2.7)	24 (2.0)
Coronavirus types OC43 and 229E	18 (1.5)	9 (0.7)
Human metapneumovirus	19 (1.5)	11 (0.9)
Patients infected with ≥ 2 viruses	266 (21.6)	/
Patients infected with ≥ 1 viruses	652 (53.1)	386 (31.4)

NOTE: Human coronaviruses, parainfluenza virus type 4, rhinovirus, human metapneumovirus, and human bocavirus 1 were studied only by PCR; respiratory syncytial virus, parainfluenza virus type 1–3, adenovirus, and influenza were studied by viral culture and PCR.

### Seasonal distribution of HBoV1

**Figure 1:**
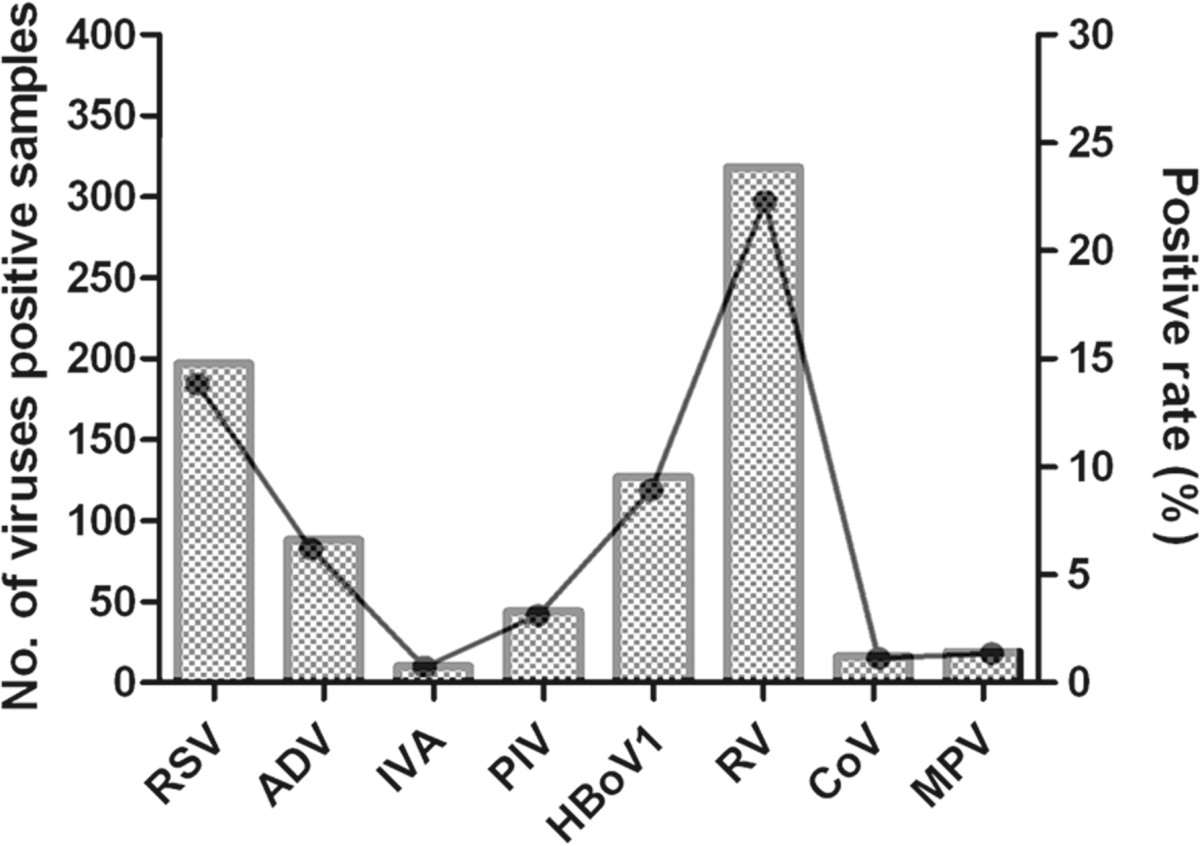
**Pathogen spectrum of 1229 patients hospitalized with respiratory tract infections.**

**Figure 2:**
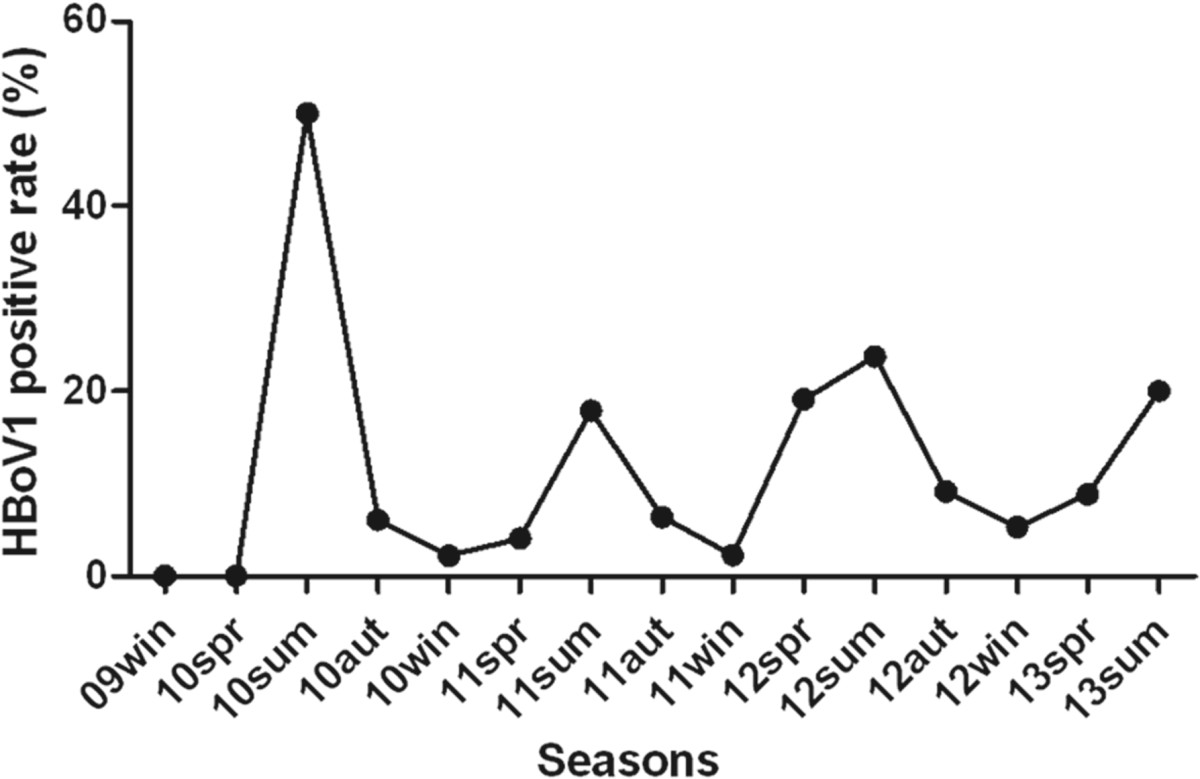
**Seasonal distribution of HBoV1 and the positive rate in each season.**

Between December 2009 and August 2013, 127 patients (10.3%) tested positive for HBoV1 in the NPAs. HBoV1 was the only virus detected in 66 patients (5.4% of all the patients and 52% of the HBoV1-positive patients). The pathogen spectrum is shown in Figure 1. Seasonal variation was observed with a high HBoV1 detection rate in summer. The seasonal distribution of HBoV1 and the positive rate in each season are shown in Figure 2.

### Quantitative analysis of HBoV1 DNA in the NPAs

**Figure 3:**
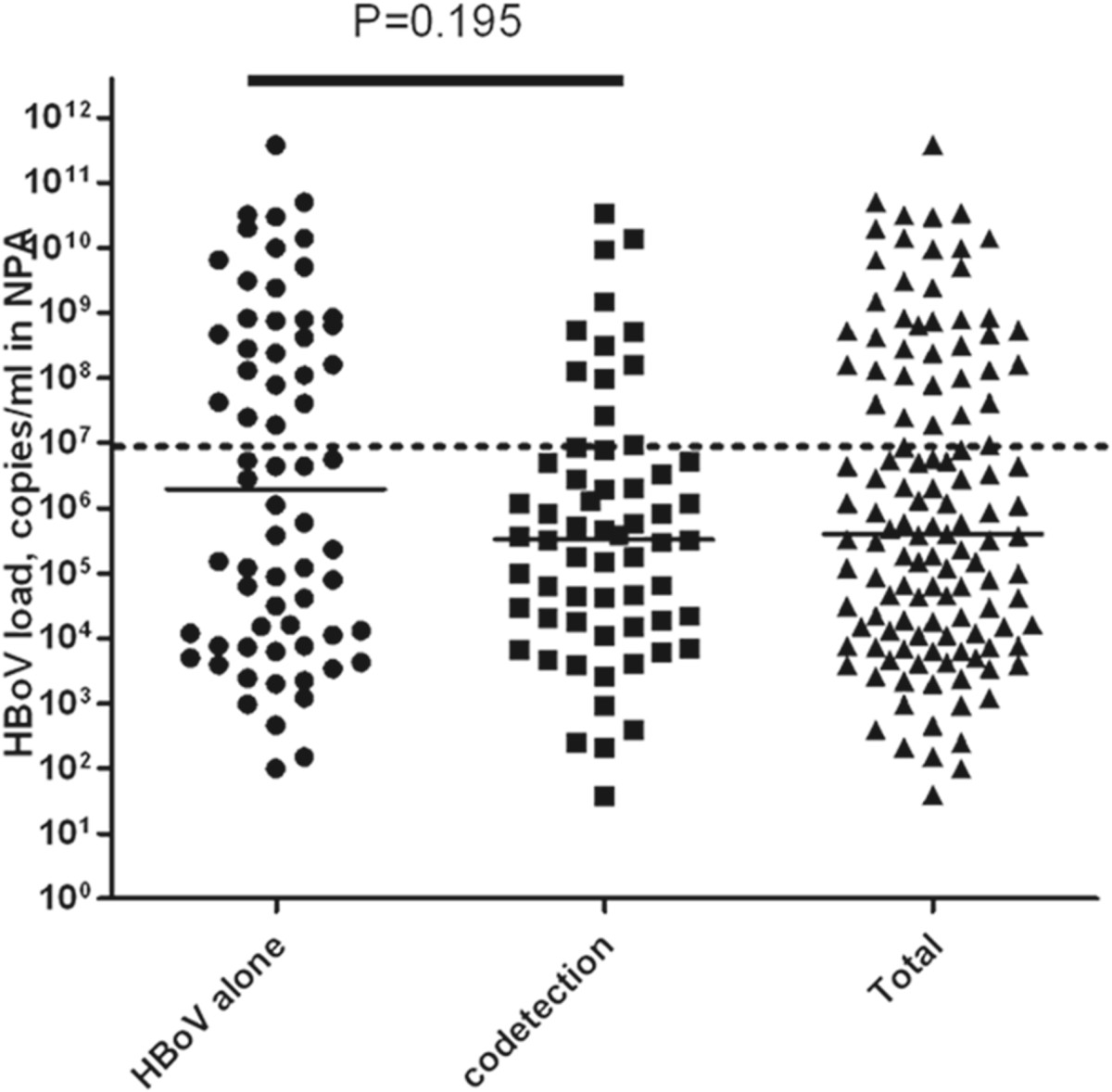
**Distribution of the HBoV1 loads among 127 nasopharyngeal aspirate samples that tested positive for HBoV1.** Each sample is represented by a single dot. The dotted line indicates the cutoff between the high and low HBoV1 load groups discussed in the text. The comparison in the mean viral load between HBoV1 high and low viral load groups were conducted by nonparametric Mann–Whitney *U*-test.

The genome viral loads in the NPAs ranged from <500 to 3.8 × 10^11^ copies/mL of the sample material. The viral loads were assigned to 2 non-overlapping populations: one group of 39 samples with a high viral load (≥ 10^7^ copies/mL) and the other group of 88 samples with a low viral load (<10^7^ copies/mL). The median levels of the HBoV1 DNA genome in the respiratory samples were higher in the patients with a single HBoV1 detection than in the patients with a mixed respiratory viral infection with HBoV1 (1.95 × 10^6^ copies/mL vs. 3.3 × 10^5^ copies/mL, P = 0.195) (Figure 3).

### HBoV1 co-detection with other respiratory viruses

HBoV1 was a mixed detection in 61 patients (48% of the HBoV1-positive patients). Of the mixed detection cases, the most frequently found concurrent co-detection with HBoV1 were RV (in 37 children), followed by ADV (in 16 children) and RSV (in 13 children). When the clinical symptoms and severity were compared, no difference was found between HBoV1 single detection and co-detection (data not shown). In the high viral load group, 71.8% of the RTI patients were detected as having HBoV1 single detection whereas 28.2% of the patients had HBoV1 co-detection with another virus. In the low viral load group, 43.2% of the patients had HBoV1 single detection, and 56.8% of the cases were HBoV1 co-detection with another virus. When compared with HBoV1-negative persons, there was a significant difference between the patients with HBoV1 single detection and the co-detection group at an HBoV1 high viral load with P = 0.017 (Table 2). These results indicated that HBoV1 at a high viral load was not frequently co-detection with other respiratory viruses.

**Table 2:** **The distribution of patients by the presence of HBoV1, the viral load in the NPAs, and the presence or absence of other viruses**

Patient group	No. of patients		P^a^
	No. (%) of children with other virus detected	No. (%) of children with no other virus detected	
HBoV1-positive patients, viral load	61 (48.0)	66 (52.0)	1.00
≥10^7^copies/mL	11 (28.2)	28 (71.8)	0.017
<10^7^copies/mL	50 (56.8)	38 (43.2)	0.097
HBoV1-negative subjects	525 (47.6)	577 (52.4)	
All subjects	652 (53.1)	577 (46.9)	

NOTE: ^a.^Compared with HBoV1-negative persons; determined by the Chi-square test.

### Association of HBoV1 and SRTI

An HBoV1 low load was found significantly more frequently than was HBoV1 with a high viral load in children. Of the HBoV1 single detection occurrences, a high viral load was more prevalent among children with dyspnea and wheezing than was a low viral load (42.9% vs. 23.7%, P = 0.036; 60.7% vs. 31.6%, P = 0.018). In clinical severity, a significant difference was recorded (25.0% vs. 5.3%, P = 0.003) between the high viral load and low viral load group (Table 3). We analyzed 18 HBoV1-positive patients who suffered from SRTI to study the association between HBoV1 and SRTI. Fourteen children had a diagnosis of severe pneumonia, 3 children had severe bronchiolitis, and 1 child had plastic bronchitis. Ten of 18 (55.6%) patients had a high HBoV1 load and only 3 of 10 children had other co-detected viruses, whereas 7 in 10 children with HBoV1 single detected were diagnosed with SRTI. The other 8 children who were diagnosed with SRTI had a low HBoV1 load. Among these 8 children, only 2 children were with HBoV1 single detected, whereas the other 6 children were co-detected with ADV, RSV or RV. HBoV1 single infection was more prevalent in the children with a high viral load than in those with a low viral load (70% vs. 25%) in the severe cases.

**Table 3:** **Comparison of demographic and clinical characteristics of patients hospitalized for HBoV1 single infection on the basis of viral loads**

	HBoV1 load <10^7^group (N = 38)	HBoV1 load ≥10^7^group (N = 28)	P value
Gender (Male)	22 (57.9)	15 (53.6)	0.727
Age (months)	11 (1–151)	9.5 (1–33)	0.846
Duration of symptoms before admission (days)	7 (1–90)	9.5 (1–30)	0.974
Duration of hospitalization (days)	7 (3–28)	7 (3–33)	0.642
Pulse rate (/min)	125 (85–168)	124 (50–180)	0.507
Respiratory rate (/min)	41 (25–58)	45 (28–67)	0.144
White blood cell (×10^9^ cells/mL)	9.6 (4–23.2)	12.6 (6.4-23.3)	0.056
Fever	20 (52.6)	16 (57.1)	0.716
Cough	34 (89.5)	27 (96.4)	0.385
Dyspnea	9 (23.7)	12 (42.9)	0.036
Rhinorrhea	8 (21.1)	3 (10.7)	0.331
Diarrhea	12 (31.6)	9 (32.1)	0.961
Wheezing	12 (31.6)	17 (60.7)	0.018
Abnormality on chest radiograph^a^	30 (100)	19 (95.0)	0.400
Severity	2 (5.3)	7 (25.0)	0.003
Upper respiratory tract infection	1 (2.6)	0 (0)	1.000
Lower respiratory tract infection			
Pneumonia	28 (73.7)	18 (64.3)	0.437
Bronchiolitis	3 (7.9)	3 (10.7)	0.693
Bronchitis	1 (2.6)	3 (10.7)	0.304
Asthma	5 (13.2)	4 (14.3)	1.000

NOTE: Data presented as median (range) or frequency (percentage) of patients. ^a.^There were 30 patients who had a chest radiograph test in the HBoV1 load <10^7^ group, and there were 20 patients in the HBoV1 load ≥10^7^group. Categorical variables were compared using the Chi-square test, and the continuous variables were compared using Student’s *t*-test or the nonparametric Mann–Whitney *U*-test.

### The clinical data of children of HBoV1 single detection at a high viral load

There were 7 children with HBoV1 single detection and a high viral load who had severe pneumonia or bronchiolitis. The demographic and clinical characteristics are shown in Table 4. The median age of the patients was 9 months (range, 3–22 months). Among the patients, there were 3 boys and 4 girls. The median hospital stay was 8 days (range, 5–33 days), and the median duration of symptoms before admission was 4 days (range, 1–15 days). Underlying conditions included congenital cardiac condition, pulmonary conditions, primary immunodeficiency or prematurity (<37 weeks), anemia and malnutrition. All the children had cough and dyspnea, 5 had fever and wheezing, 3 had diarrhea and 2 children received treatment in ICU. The patient in case 1 had right lower pulmonary atelectasis and required ICU treatment for 25 days, with ventilator assisted breathing for 7 days. The patient in case 7 remained in ICU for 8 days, with ventilator assisted breathing for 5 days. Chest radiography showed 6 cases with pulmonary inflammation (one patient did not have a chest radiography test). Only one case (case 1) had complications, and the prognosis was positive; 4 children were discharged after 5–12 days of hospitalization whereas the other 3 children were improving after 8–33 days of hospitalization and subsequently recovered.

**Table 4:** **Seven cases with human bocavirus1 single infection at high viral load associated with severe respiratory tract infections**

	Case 1	Case 2	Case 3	Case 4	Case 5	Case 6	Case 7
HBoV1 viral load (copies/mL)	3.8 × 10^11^	7.9 × 10^8^	7.4 × 10^8^	2.5 × 10^7^	6.5 × 10^9^	5.1 × 10^9^	8.4 × 10^8^
Gender	Female	Male	Female	Male	Female	Female	Male
Age (months)	10	3	9	22	9	9	12
Underlying disease	With mild anemia	No	Born at 34 weeks	No	Born at 28 weeks	Born at 35 weeks With mild anemia	No
Duration of symptoms before admission (days)	7	9	15	1	3	4	3
Duration of hospitalization (days)	33	12	6	5	7	8	20
Fever	Yes	Yes	No	No	Yes	Yes	Yes
Cough	Yes	Yes	Yes	Yes	Yes	Yes	Yes
Rhinorrhea	No	No	Yes	No	Yes	No	Yes
Wheezing	Yes	No	Yes	Yes	No	Yes	Yes
Dyspnea	Yes	Yes	Yes	Yes	Yes	Yes	Yes
Diarrhea	No	Yes	No	No	Yes	Yes	No
Weight on admission (kg)	6.0	7.3	6.0	11.0	9.0	7.0	8.0
Body temperature (°C)	38.8	37.7	37.0	37.0	38.4	37.4	39.1
Respiratory rate (/min)	30	67	58	32	50	49	62
Pulse rate (/min)	124	180	145	50	160	145	148
White blood cell (×10^9^ cells/mL)	10.3	15.3	20.6	14.3	12.7	11.8	8.9
Neutrophil granulocyte %	61	26	34	85	68	32	68
Hemoglobin (g/l)	97	107	120	125	120	94	106
Lymphocyte %	35	70	61	12	25	67	26
Platelets (10^9^/L)	354	420	457	352	273	499	265
Chest radiograph	Yes^a^	Yes^b^	Yes^c^	-	Yes^b^	Yes^b^	Yes^d^
Abnormality in blood culture	No	No	No	No	No	No	No
Bacterium in sputum culture	E. coli bacteria	Geleibai coli pneumonia	Streptococcus pneumoniae and Haemophilus influenzae	No	No	No	Streptococcus pneumoniae
Entering the intensive care unit^e^	Yes	No	No	No	No	No	Yes
Complication	The right lower pulmonary atelectasis	No	No	No	No	No	No
Final diagnosis	Severe pneumonia	Severe bronchiolitis	Severe pneumonia	Severe pneumonia	Severe pneumonia	Severe pneumonia	Severe pneumonia
Prognosis	Recover	Cure	Cure	Cure	Cure	Recover	Recover

Note: ^a^Double lung inflammation, right lower pulmonary atelectasis; ^b^Double lung texture fuzzy, with visible flocculent shadow and lung inflation is a bit excessive; ^c^Double lung inflammation, light transmittance in left lung is higher than the right lung; ^d^Double lung lesions, the consolidation of the right lung is obvious. ^e^The patient in case 1 received ICU treatment for 25 days and ventilator assisted breathing for 7 days. The patient in case 7 received ICU treatment for 8 days and ventilator assisted breathing for 5 days.

## Discussion

This study confirms that HBoV1 is frequently found in children with RTI. We conducted this study for nearly 4 consecutive years, and samples were analyzed for 15 respiratory viruses by PCR and viral culture. The HBoV1 viral load was quantified by real-time fluorescence quantitative PCR. The results suggest that HBoV1 at a high viral load (≥10^7^ copies/mL) is not co-detected frequently with other respiratory viruses; there would be an association between HBoV1 single detection at a high viral load and SRTI.

This study investigated the role of HBoV1 in RTI and found that 10.3% of the children with RTI were HBoV1 positive in the NPAs. Multiple studies have confirmed the presence of HBoV1 in respiratory tract samples of children world wide [7, 21, 22]. Some reports indicate that HBoV1 infection is associated with acute respiratory tract symptoms and that a high HBoV1 load (≥10^7^ copies/mL) is associated with SRTI [14, 23]. In this study, HBoV1 low load was found significantly more frequently in children than was HBoV1 with a high viral load. The cases of SRTI in the patients with HBoV1 detection alone appeared to be particularly common among children with a high HBoV1 load. The stratification of the patients on the basis of the HBoV1 viral load ≥10^7^ copies/mL versus <10^7^ copies/mL in NPAs revealed that the presence of HBoV1 single detection at high (≥10^7^ copies/mL)—but not at low (<10^7^ copies/mL)—viral loads is associated with SRTI. Similarly, a case report described HBoV detection leading to SRTI [24]. Zhao found an association between disease severity and the HBoV1 viral load in co-detection children [14]. Our study reported an association between the HBoV1 high viral load and SRTI in single detection children. We confirmed HBoV1 single detection at a high viral load in 7 children. Two of them received treatment in ICU, including ventilator assisted breathing. These cases demonstrate that a lower respiratory tract infection caused by HBoV1 single detection at a high viral load could lead to a severe and life-threatening disease.

In most of the previous studies, the co-detection rate ranged from 18-72% [25]. In this study, of the HBoV1 positive samples, 48% were co-detected with other respiratory viruses (HBoV1-RV in 37 children, followed by HBoV1-ADV in 16 children and HBoV1-RSV in 13 children). This result is consistent with more recent studies. Martin reported that 35.3% of HBoV cases were co-infected, and 83.3% of them were HBoV-RSV co-infection [7]. Another study reported that RSV and RV were most frequently detected in conjunction with HBoV1 [26], and ADV and enterovirus were more common in children with HBoV [5]. We compared HBoV1 single detection and co-detection, and the median level of HBoV1 load was greater in the subjects with HBoV1 infection alone than in the subjects with mixed respiratory viral infections. There were no differences in the clinical symptoms and severity. Co-detection with HBoV1 did not increase the severity of RTI. High viral loads of HBoV1 were noted predominantly in the absence of other viral agents, whereas co-detections were primarily found in low viral load cases. In severe cases, HBoV1 single detection was more prevalent among the children with a high viral load than those with a low viral load.

Recently, some studies reported that HBoV was likely to persist in respiratory samples of asymptomatic patients [27, 28]. Cashman also showed that HBoV1 was found in stool samples of asymptomatic patients [29]. Allander suggested a model for HBoV infection in which high viral loads are potentially associated with respiratory symptoms, and low viral loads indicate asymptomatic shedding [3]. In our study, a portion of patients with HBoV1 single detections at low viral load suffered from acute respiratory tract disease. What’s more, Schildgen suggested that HBoV may indirectly contribute to the development of some colorectal and lung cancers or may play an active role in cancer by interacting with the host genome [30]. Thus, it is hard to make a conclusion that a portion of HBoV single detections with low copy number is always accompanied by a lack of symptoms. The role of HBoV is sometimes as a passenger and sometimes as a pathogen in acute respiratory tract disease.

One of the limitations in this report is that this study included only hospitalized patients with RTI and was lack of a control group (individuals without respiratory symptoms). Secondly, we did not detect the HBoV1 DNA load and culture HBoV1 in the serum samples and serological analysis for IgM and IgG. The most reliable methods for diagnosis of acute symptomatic HBoV infections are PCR of serum samples and serological analysis for IgM and IgG [31]. Christensen suggested that, for clinical purposes, HBoV mRNA is more accurate than HBoV DNA in diagnosing active HBoV infection; however, a high HBoV DNA load (>10^7^ copies/mL) might be useful for the diagnosis [8].

## Conclusion

In conclusion, HBoV1 is a prevalent virus commonly detected in hospitalized children with RTI especially in children below 2 years of age. During the study period, the high detection of HBoV1 was predominantly in May-August and November-January. Of the HBoV1 single detection cases, the high viral load group was more prevalent than the low viral load group among the children with dyspnea and wheezing. The disease severity in the high viral load group was greater than the severity of the low viral load group. The 7 cases reported here suggest that a lower respiratory tract infection caused by HBoV1 single detection at a high viral load (≥10^7^ copies/mL) could lead to severe and life-threatening disease. HBoV1 could in some cases be a passenger in RTI, primarily in the low viral load group. HBoV1 co-detection with other respiratory viruses did not increase the severity of RTI. However, the role of HBoV1 detection in SRTI merits further study.

## Authors’ original submitted files for images

Below are the links to the authors’ original submitted files for images. Authors’ original file for figure 1 Authors’ original file for figure 2 Authors’ original file for figure 3

## Abbreviations

ADV: Adenovirus
CoV: Coronaviruses
HBoV1: Human bocavirus1
ICU: Intensive care unit
IVA: Influenza virus A
IVB: Influenza virus B
IVC: Influenza virus C
MPV: Metapneumovirus
NPAs: Nasopharyngeal aspirates
PCR: Polymerase chain reaction
PIV1–4: Parainfluenza virus types 1–4
RSV: Respiratory syncytial virus
RTI: Respiratory tract infections
RV: Rhinovirus
SRTI: Severe respiratory tract infections.
