# Supplementary figures and images for: Single detection of human bocavirus 1 with a high viral load in severe respiratory tract infections in previously healthy children
Source: BMC Infect Dis. 2014 Jul 30;14:424. doi: 10.1186/1471-2334-14-424 (PMC4125703; doi:10.1186/1471-2334-14-424)

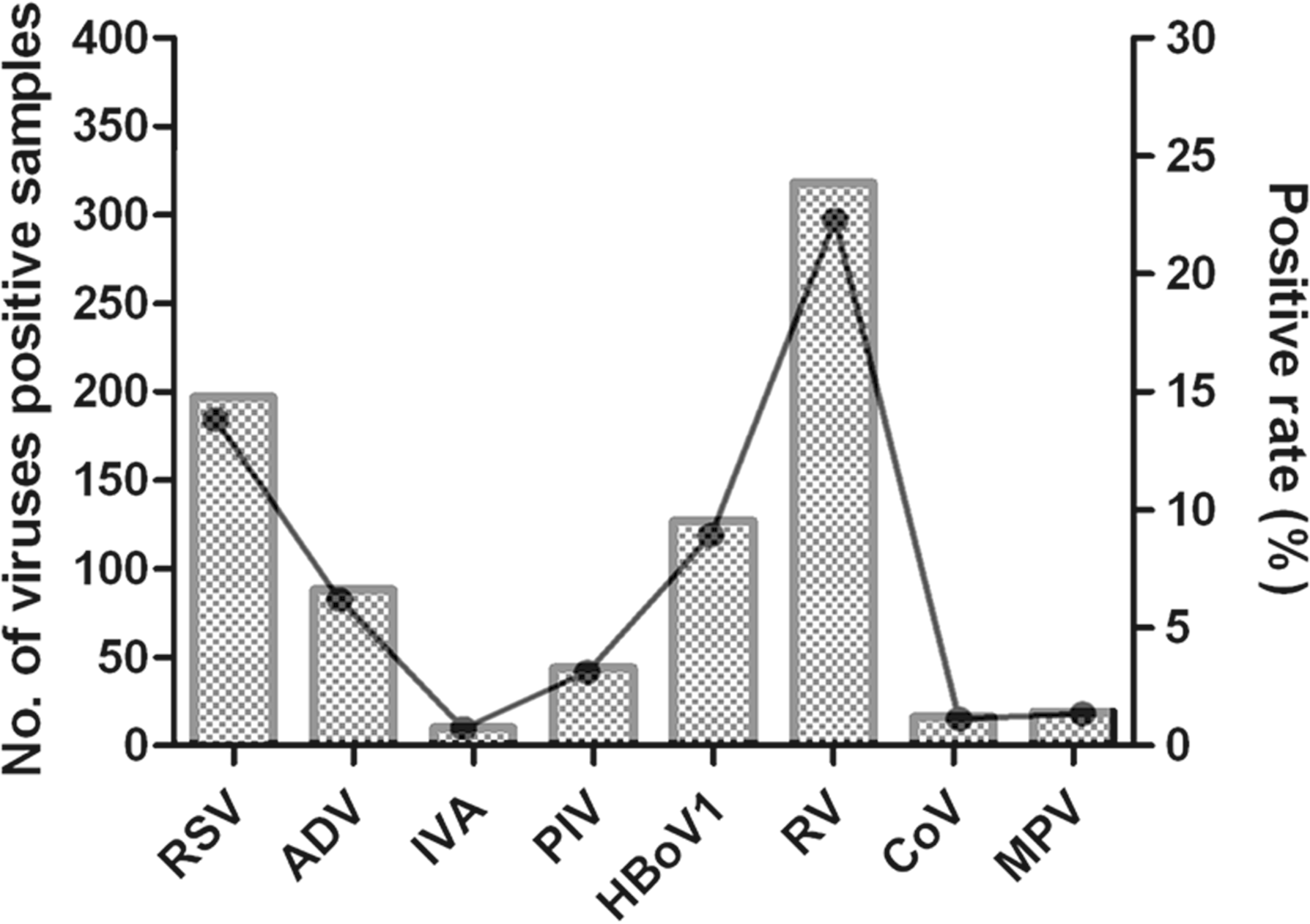

Supplement: Supplementary file 1 — Authors’ original file for figure 1 [file 12879_2014_3723_MOESM1_ESM.tif]

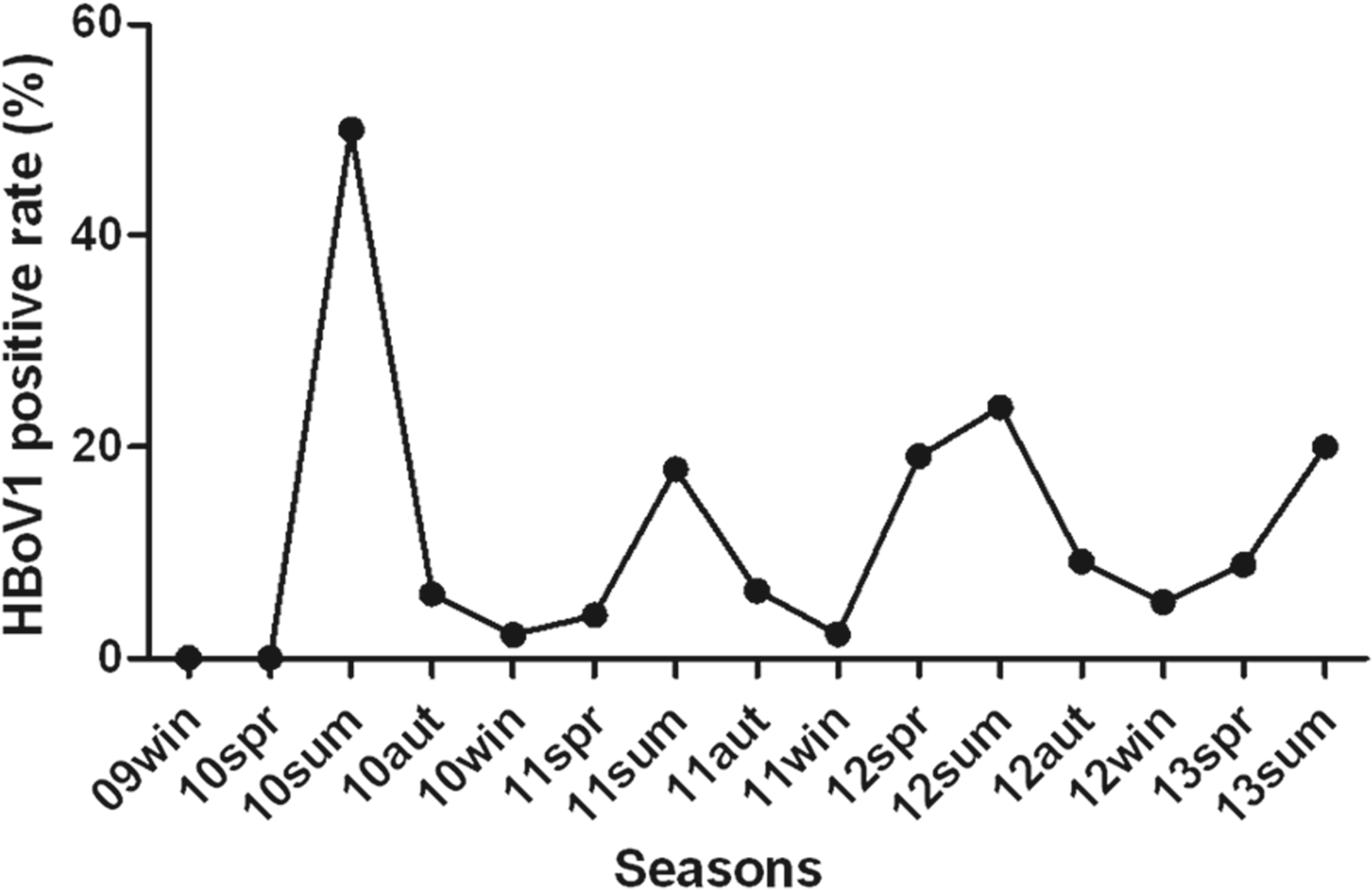

Supplement: Supplementary file 2 — Authors’ original file for figure 2 [file 12879_2014_3723_MOESM2_ESM.tif]

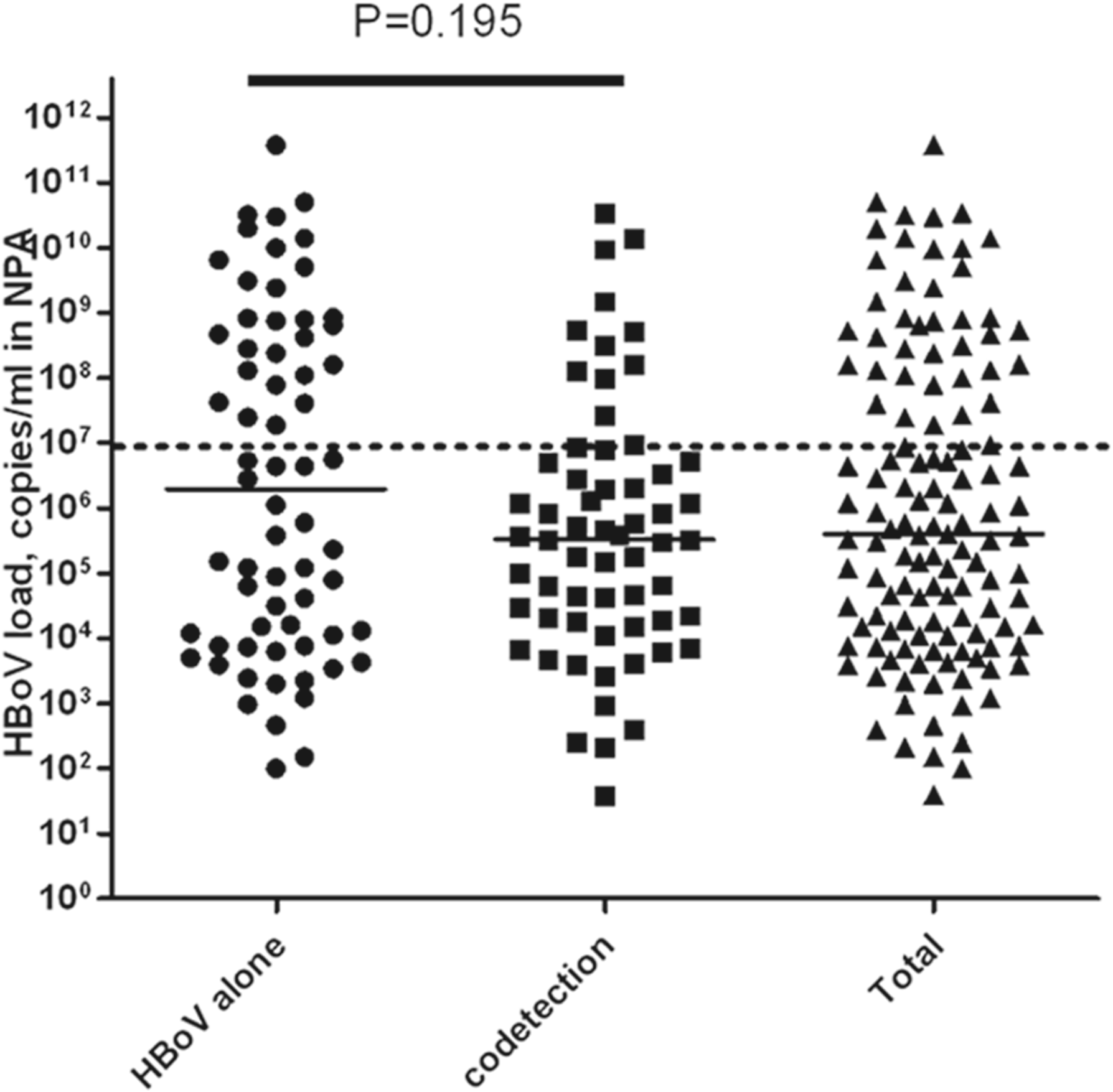

Supplement: Supplementary file 3 — Authors’ original file for figure 3 [file 12879_2014_3723_MOESM3_ESM.tif]
